# Rhinovirus Infects B and CD4 T Lymphocytes in Hypertrophic Tonsils in Children

**DOI:** 10.1002/jmv.70809

**Published:** 2026-01-24

**Authors:** Ronaldo Martins, Flavia E. de Paula, Talita B. G. Mitchell, Miria F. Criado, Ricardo S. Cardoso, Bruna L. S. Jesus, Italo A. Castro, Murilo Henrique Anzolini Cassiano, Daniela Méria Ramos Rodrigues, Noilson Oliveira, Lucas Carenzi, Fabiana C. Valera, Edwin Tamashiro, Wilma T. Anselmo‐Lima, Eurico Arruda

**Affiliations:** ^1^ Department of Clinical Analyzes, Toxicology and Food Science (DACTB) University of São Paulo School of Pharmaceutical Sciences of Ribeirao Preto Ribeirão Preto Brazil; ^2^ Department of Cell Biology and Virology Research Center University of São Paulo School of Medicine Ribeirão Preto Brazil; ^3^ Institute of Mathematics and Statistics University of São Paulo São Paulo Brazil; ^4^ Department of Ophthalmology, Otorhinolaryngology and Head and Neck Surgery University of Sao Paulo School of Medicine Ribeirão Preto Brazil

**Keywords:** adenotonsillar hypertrophy, cytokines, lymphocytes, rhinovirus, tonsillectomy

## Abstract

Prolonged detection of rhinovirus (RV) in secretions after a typical cold and asymptomatic shedding are frequently reported. Although RV has been detected in human hypertrophic tonsils, its replicative status and host cell range remain unclear. In this study, we analyzed RV replication, infected cell types, and recovery of infectious virus in adenoids, palatine tonsils, and respiratory secretions from 293 children with tonsillar hypertrophy undergoing tonsillectomy. Samples were screened by real‐time RT‐PCR, and RV‐positive samples were analyzed using immunohistochemistry (IHC), chromogenic in situ hybridization (CISH), flow cytometry, and RV isolation in cell culture. RV genotypes from species A, B, and C were identified in adenotonsillar samples. RV antigenome and structural proteins were detected in tonsillar epithelial surfaces, parenchyma, and in CD4 + T and B lymphocytes. Infectious RV was recovered from adenoids and respiratory secretions. In vitro infection of tonsillar mononuclear cells with RV‐16 and RV‐1A resulted in viral progeny production and secretion of distinct cytokine profiles. These findings demonstrate that RV infects tonsillar T and B lymphocytes, suggesting that tonsils can serve as sites of prolonged infection and sources of RV shedding. RV infection of immune cells may have potential impact on the local immune microenvironment.

## 1 Introduction

Rhinovirus (RV) is the most frequent cause of common cold, a mild acute respiratory infection (ARI) often complicated by asthma exacerbation, acute otitis media, or sinusitis [1, 2]. We have previously reported the detection of RV RNA by real‐time polymerase chain reaction (RT‐PCR) in 25% of tissues removed from adenoids, 12% from palatine tonsils, and 35% in nasopharyngeal secretions (NPS) from children with chronic tonsillar hypertrophy undergoing adenotonsillectomy, in the absence of ARI symptoms [3]. Similar findings were later reported by other groups [4, 5]. However, these earlier studies focused predominantly on viral RNA detection, and did not establish whether the virus was actively replicating, which cell types were infected, or whether infectious virus could be recovered from these tissues. Moreover, the immunological implications of RV infection in tonsillar lymphocytes—particularly CD4⁺ T and B cells—remain largely unexplored.

In this study, we aimed to fill these knowledge gaps by analyzing not only the presence of RV genomes but also the replicative status of the virus, the phenotypes of infected cells, and the recovery of infectious RV from tonsillar tissues and respiratory secretions of a large cohort of children (*n* = 293) undergoing tonsillectomy. Our findings demonstrate active RV replication in tonsillar tissues, including in B and CD4⁺ T lymphocytes, and provide compelling evidence that both adenoids and palatine tonsils can act as reservoirs for infectious RV. In several patients, the same infectious RV genotypes were simultaneously identified in adenoids, palatine tonsils, and nasopharyngeal secretions, supporting the role of tonsils as potential viral reservoirs. Furthermore, RV infection of tonsillar lymphocytes resulted in the production of infectious progeny and triggered distinct pro‐inflammatory cytokine responses, including elevated levels of IL‐17, TNF‐α, and IFN‐γ, suggesting that RV may modulate the local immune microenvironment within lymphoid tissues. Collectively, the findings suggest that tonsils could be reservoirs of RV. This represents a novel insight into the biology of RV, with potential implications for understanding prolonged shedding, virus persistence, and local immune modulation.

## 2 Methods

### 2.1 Study Design and Sample Processing

This prospective cross‐sectional study enrolled 293 children (54.6% boys) 3 to 13 years of age (median 6 years; mean 5 years) who underwent adeno‐tonsillectomy due to tonsillar hypertrophy or recurrent tonsillitis, at the Division of Otorhinolaryngology, Clinical Hospital of the University of São Paulo School of Medicine, in Ribeirão Preto, Brazil. Exclusion criteria were the presence of ARI symptoms or antibiotic treatment within 1 month before surgery. Informed consents were obtained from parents/guardians in compliance with the Human Research Ethics Committee of the University of Sao Paulo Clinical Hospital, Ribeirão Preto (number 10466/2008). This was an exploratory study aimed at characterizing rhinovirus infection in tonsillar tissues. Mucosal swabs were collected from the regions of adenoids and palatine tonsils under direct view after anesthesia, and fragments of tonsillar tissues were obtained during surgery. Specimens were transported on ice in less than 2 h to the virology laboratory, where they were processed and tested by real‐time polymerase chain reaction (RT‐PCR) for RV. Briefly, fragments of tissues of approximately 1 g including epithelial surface and parenchyma were preserved in RNAlater (Invitrogen, Carlsbad, CA, USA) for nucleic acid extraction. From the same tissues, a second similar‐size fragment was placed in freezing medium for later virus isolation, another was dissociated by enzymatic digestion to purify tonsillar mononuclear cells (TMNCs), and yet another was fixed and paraffin‐embedded for histology.

TMNCs were prepared by tissue digestion with dispase (0.6 U/mL) and collagenase‐I (100 U/mL) (both from Gibco, Grand Island, NY, USA). The fragments destined to histology were fixed for 4 h in Carnoy's fixative, composed of 60% ethanol, 30% acetic acid, and 10% chloroform (Merck, Darmstadt, Germany), then dehydrated in ethanol series (JT Baker, Mexico) for 1 h each (70%, 80%, 90%, 95%, and three changes of 100%), followed by 1:1 xylene:ethanol for 20 min, and three 30‐min changes of pure xylene (Synth, Diadema, SP, Brazil). The tissues were then paraffin‐embedded by dipping in two changes of paraffin (Merck) for 2 h each, and the blocks were used to prepare 3 µm sections on positively charged slides (Fisher, Pittsburgh, USA). Nasopharyngeal swabs (NPS) were eluted in PBS and the eluate was split for extraction by Trizol and for freezing at −80°C until further testing.

A schematic flowchart summarizing the sample collection and processing workflow is presented in Supporting Information Figure 1.

### 2.2 Detection of RV Genomes and Genotyping of RV Species

The detection of RV and EV genomes was performed using TaqMan real‐time RT‐PCR, with total nucleic acids extracted via Trizol® from 250 µL of homogenized tissue samples or swab eluates, in accordance with protocols previously published by our group [3, 6]. Specific primers and probes for RV and EV, as well as for the housekeeping genes β‐actin and RNase P, are listed in Table 1. Reverse transcription was done on 1 µg of extracted RNA using random hexamers and Multiscribe Reverse Transcriptase (Applied Biosystems, Foster City, CA, USA). All real‐time PCR assays were done on a Step‐One Plus real‐time PCR thermocycler (Applied Biosystems, Foster City, CA, USA) in a final volume of 15 µL using 3 µL of cDNA, 10 µM forward and reverse primers, 5 µM probe, 0.15 µL of Rox and 7.5 µL of TaqMan master mix (Sigma‐Aldrich, St. Louis, MO, EUA). The cycling was 95°C for 3 min, followed by 45 cycles of 95°C for 10 s, and 60°C for 30 s, with final soaking at 10°C. Real‐time PCR for enterovirus (EV) was done in a final volume of 10 µL, with 3 µL of cDNA, 10 µM forward and reverse primers, 5 µM probe, 5 µL of TaqMan master mix (Applied Biosystems, Foster City, CA, USA), with cycling at 95°C for 10 min, followed by 45 cycles of 95°C for 15 s, and 60°C for 1 min. RV genotyping was done in the RV‐positive samples by conventional RT‐PCR using primers targeting conserved sequences in the 5′UTR of the RV genome, followed by sequencing of the PCR products [1]. Briefly, RV amplicons to be sequenced were generated by two sequential amplification steps, following a previously published protocol and a mix of three forward primers (B1‐3) plus one reverse primer R2 (Table 1). Reactions were done at the final concentration of 25 μM, with the enzyme Platinum PCR supermix HF (Invitrogen), on 100 ng of cDNA, according to the following parameters: touch‐down with two cycles of incubation at 94°C for 2 min and 20 s, plus 68°C for 70 s. The cycling was repeated eight times at sequential annealing temperatures of 66°C, 64°C, 62°C, 60°C, 58°C, 56°C, 54°C, and 52°C for two cycles each, and a final 10‐min extension at 68°C. After the first round of PCR, a second round was performed using 2.5 µL of the product of the first round, with the same primers, using the following parameters: 94°C for 2 min, 94°C for 20 s, 52°C for 30 s, and 68°C for 40 s, with 27 repetitions and a final extension at 68°C for 3 min. The PCR products were treated with ExoSAP‐IT® (Affymetrix, Santa Clara, CA, EUA) with incubation at 37°C for 25 min, and then at 90°C for 15 min. The sequencing reaction was carried out with 3 μL of the PCR product using 2 μL of BigDye Terminator v3.1 Cycle Senquencing RR‐100 (Applied Biosystems, Foster City, CA, EUA), 10 pmol of the reverse primer R2 and 3 μL of buffer, at the final volume of 20 μL, in an ABI Hitachi 3500 Genetic Analyzer (Applied Biosystems, Foster City, CA, EUA). All sequences were analyzed by DNASTAR Lasergene Genomics software and the FASTA sequences were used in a BLAST search using the cut‐off parameters of identity higher than 95% and e‐value higher 10⁻⁵.

**Table 1:** Primers and probes used for real‐time PCR and for direct sequencing of polymerase chain reaction fragments of 5′UTR.

	Primer	Sequence (5′/3′)	Ref
	Fw	GCGGAACCGACTACTTTGGG	
EV	Rev	CTCAATTGTCACCATAAGCAGCC	[3]
	Probe	Fam‐TCCGTGTTTCCTTTTATTCTTATA‐MGB	
	Fw	ACMGTGTYCTAGCCTGCGTGG C	
RV	Rev	GAAACACGGACACCCAAAGTAGT	[6]
	Probe	Fam‐TCCTCCGGCCCCTGAAT‐BHQ1	
	Fw	CCCAGCCATGTACGTTGCTA	
β‐actin	Rev	TCACCGGAGTCCATCACGAT	[3]
	Probe	Fam‐ACGCCTCTGGCCGTACCACTGG‐Tamra	
	Fw	AGATTTGGACCTGCGAGCG	
RNAseP	Rev	GAGCGGCTGTCTCCACAAGT	[6]
	Probe	Fam‐TTCTGACCTGAAGGCTCTGCGCG‐BHQ1	
	HRVB1	CAAGCACTTCTGTTTCCCA	
5′UTR	HRVB2	CAAGCACTTCTGTTACCCC	[1]
	HRVB3	CAAGCACTTCTGTCTCCCC	
	FR2	ACGGACACCCAAAGTAG	

### 2.3 Serial Immunohistochemistry (SIMPLE)

To enhance the detection of multiple rhinovirus genotypes in naturally infected samples, a 1:1 antibody blend (by volume and final concentration) of anti‐VP2 (mabR16‐7, QED Bioscience, San Diego, CA, USA) and anti‐VP1 (mab8430, Millipore, Temecula, CA, USA) was used at a final dilution of 1:1000 in PBS/BSA containing 0.1% Triton X‐100 (Sigma, St. Louis, MO, USA). This antibody combination was chosen based on the manufacturer's specifications and our own validation experiments. Specifically, mabR16‐7 was confirmed to selectively detect RV‐16 and RV‐1A, whereas mab8430, originally developed against enterovirus VP1, displayed cross‐reactivity with stocks of RV‐14, RV‐39, RV‐16, and RV‐1A maintained in our laboratory. The rationale for using this blend was to increase the detection across rhinovirus genotypes frequently observed in clinical specimens, acknowledging the potential for cross‐reactivity with other enteroviruses. To minimize this, antibody blends used for IHC were applied only to samples that tested positive for RV by real‐time PCR, but not for enteroviruses. Tissues positive for both RV and enteroviruses by PCR were excluded from IHC analysis. Tissue sections were then incubated with horse anti‐mouse biotinylated antibody (BA‐2000, Vectastain ABC Kit, Vector Laboratories, Burlingame, CA). Signal amplification was attained by incubation with polymer conjugated with streptavidin‐peroxidase (s2438, Sigma‐Aldrich), and color development was done with AEC peroxidase system (SK‐4800, Vector Laboratories, Burlingame, CA), resulting in red‐purple staining in positive cells, followed by tissue counterstaining with Harris hematoxylin (Vector) [7]. To determine the types of RV‐infected lymphomononuclear cells, sequential immunoperoxidase labeling and erasing (SIMPLE) [8] was done with the above‐mentioned MAbs for virus structural protein, followed by staining with rabbit polyclonal antibodies for CD3, CD4 (AB 133616, dilution 1:100, Abcam), CD8 (AB 4055, dilution 1:100, Abcam), CD20 (AB 27093, dilution 1:100, Abcam), CD11c (AB‐52632, dilution 1:100, Abcam). Goat anti‐rabbit biotinylated antibody (ab64256, Abcam) was used as a secondary antibody for the immune phenotype staining, and color development was generated with the Vector AEC peroxidase system (SK‐4200, Vector Laboratories, Burlingame, CA). After counterstaining with Harris hematoxylin, slides were mounted with coverslips with an aqueous mounting medium. After whole‐slide high‐resolution scanning, coverslips were removed in distilled water and slides were dehydrated through an ethanol gradient to 95% ethanol. Slides were then incubated in ethanol series until the complete erasing of the AEC color. Following rehydration, previous antibodies were eluted by incubation of the tissue sections in 0.15 mM KMnO₄/0.01 M H₂SO₄ solution for 2 min, immediately followed by a distilled water wash. The same tissue sections were then stained for another antigen, beginning at the blocking step. RV‐infected and uninfected HeLa cells were included as positive and negative controls in all tested batches.

### 2.4 Chromogenic In Situ Hybridization

Chromogenic in situ hybridization (CISH) was adapted from published procedures [9]. Using a locked nucleic acid (LNA) probe consisting of a sense oligonucleotide (Dig/GCACTTCTGTTTCCCC/Dig) targeting the RV negative‐strand replicative intermediate anti‐genome RNA at a sequence within the 5′UTR. The tissues were deparaffinized in three serial xylene baths (Synth) for 5 min each, and then rehydrated in decreasing concentrations of ethanol (100%, 96%, and 70%) (JTBaker), in two changes of each concentration, the first for 1 min, and the second for 5 min. The sections were then washed for 5 min in PBS and incubated with 10 µg/mL proteinase K (Invitrogen) for 10 min at 37°C, re‐washed in two changes of PBS, and dehydrated in 70%, 96%, and 100% ethanol, twice for 1 min in each concentration. Next, the slides were air‐dried and incubated at 50°C for 1 h with a hybridization solution consisting of 4X SSC, 10 mg/mL BSA, 30% dextran, 20 U RNAse inhibitor (Fermentas, Lithuania), and 40 nM OL26 probe. The slides were washed twice in 5X SSC (50°C), twice in 1X SSC, twice in 0.2X SSC (50°C) for 5 min each, and then washed once in 0.2X SSC for 5 min at room temperature (RT). The slides were then incubated with a blocking solution (PBS pH 7.2, 0.1% Tween 20, 1% BSA, and 2% goat serum), with anti‐digoxigenin antibody (Roche, Indianapolis, USA) diluted 1:100 in PBS 1X for 1 h at RT, and washed three times for 3 min each with PBS with 0.01% Tween 20. The slides were then rinsed with 100 mM Tris‐HCl pH 9.5, incubated with the black chromogen kit for alkaline phosphatase (Vector, Burlingame, CA), counterstained with Harris hematoxylin (Vector), dehydrated, and mounted under coverslip with aqueous medium.

### 2.5 Flow Cytometry

The types of TMNCs were determined by flow cytometry using the antibodies CD3‐PE‐CF594 (562406), CD4‐PerCP‐Cy5.5 (560650), and CD20‐PE‐Cy7 (560735), all acquired from BD Horizon (Franklin Lakes, NJ, USA). Internal flow cytometry controls were done by incubating RV‐infected cells with the fluorescence‐conjugated secondary antibody in the absence of primary antibody. Cell suspensions were fixed with 4% paraformaldehyde for 20 min, followed by incubation with permeabilization buffer (0.1% saponin in PBS) for 5 min, and then with blocking solution (5% BSA in PBS) for 30 min. For the staining of viral structural protein, cells were incubated with a blend of anti‐VP2 (mabR16‐7) and anti‐VP1 (mab8430) diluted 1:300, for 1 h at RT, followed by three washes in PBS, and then incubated with FITC‐labeled secondary goat anti‐mouse IgG Apl24F antibody (Millipore, Temecula, CA, USA) for 30 min at RT. Flow cytometry was performed on a Becton Dickinson 6 FACS‐Canto machine, and data analyzes were done with FlowJo version 9.4.3, after appropriate gating on CD3^+^/CD4^+^ and CD20^+^ populations. As previously stated, a 1:1 blend of anti‐VP2 (mabR16‐7) and anti‐VP1 (mab8430) antibodies was used to enhance detection sensitivity across a broader range of RV genotypes. Flow cytometry data were acquired from three independent experiments, with a minimum of 20,000 gated events per sample to ensure statistical robustness.

### 2.6 Rhinovirus Isolation

Isolation of RV from tissue fragments and NPS was attempted in HeLa‐I and WI‐38 cells, based on published procedures [10]. Briefly, tissue homogenized on TissueLyser LT (Qiagen) was snap‐frozen in liquid nitrogen and then thawed, clarified by centrifugation (10 min at 1000*g* at 4°C), and filtered through 0.22 µm filter. Filtered and clarified NPS or tissue lysates were inoculated onto 75% confluent monolayers of HeLa‐I or WI‐38 cells in 24‐well tissue culture plates, and incubated at 33°C in 5% CO_2_ in McCoy's medium (Sigma‐Aldrich) with 2% FBS supplemented with 30 mM MgCl_2_ and antibiotics. Infected monolayers were monitored daily and harvested when cytopathic effect (CPE) was advanced, and RV isolation was confirmed by immunofluorescence (IF) with a blend of mouse monoclonal antibodies anti‐VP2/anti‐VP1. Briefly, cells from CPE‐positive monolayers were spotted onto glass slides, fixed in cold acetone for 5 min, and stored at −20°C until IF‐testing. Slides were defrosted and incubated with a permeabilizing/blocking solution (PBS with 0.01% Triton, 1% BSA Fraction V (Sigma‐Aldrich), and 5% goat serum for 5 min at RT. Slides were washed in PBS, incubated with anti‐VP2/anti‐VP1 diluted 1:300 in PBS with 1%BSA at RT for 30 min, washed three times with PBS and incubated with FITC‐labeled goat anti‐mouse IgG antibody Apl24F (Millipore, Temecula, CA, USA) diluted 1:100 in PBS, for 30 min at room temperature protected from light, and then washed three times with PBS. Slides were incubated for 5 min at RT with DAPI (1 µg/ml) for nuclei staining, and then mounted in mounting media (Dako) to be examined in a fluorescence microscope. Cross‐reactivity of anti‐VP1 EV blend with RV was confirmed using major and minor types RV‐16 and RV‐1A (Supporting Information Figure 2). Samples that did not cause CPE in cultures were subject to 3 additional blind passages in fresh monolayers before being discarded as negative.

### 2.7 Infection of TMNCs With RV In Vitro

TMNCs prepared from tonsils negative for RV and EV by RT‐PCR were obtained by enzymatic dissociation of tonsillar tissue fragments with dispase‐collagenase and Ficoll‐Paque Plus® (Merck Millipore, EUA) density‐gradient centrifugation. Briefly, RV‐ and EV‐negative TMNCs were inoculated in suspension (MOI = 1) by incubation for 2 h at 33°C with RV‐16, RV‐1A, respectively major and minor RV genotypes, or mock‐inoculated with extracts of uninfected HeLa cells. The MOI of 1 was chosen to maximize the exposure of the heterogeneous TMNC population, and enable the detection of viral replication and cytokine response within a short period of time post infection. After inoculation, TMNCs were washed in the Hanks' balanced salt solution (HBSS) buffer, resuspended in RPMI, and cultured for 24 h at 33°C in 5% CO_2_. Virus titers were determined in cells and supernatants by TCID_50_ in HeLa cells at 2, 4, 6, 8, 12, 16, and 24 h post‐infection. The cytokines IL‐2, IL‐4, IL‐6, IL‐10, TNF, IFNγ, and IL‐17A were quantified in the supernatants from RV‐infected TMNCs using a human cytometric bead array (CBA) Th1/Th2/Th17 Cytokine Kit (BD Biosciences) following the manufacturer's instructions, using BD Accuri C6 Plus Personal Flow Cytometer.

## 3 Results

### 3.1 The Detection and Typing of RV in Adenoids and Palatine Tonsils

Different RV genotypes were detected by RT‐PCR in at least one sampling site from 137 of 293 (46.7%) patients. Overall, RV was detected more frequently in adenoids (97 of 293; 33%) than palatine tonsils (74 of 293; 25.2%), but the difference was not significant (*p*‐value 0.9820). RV was detected in 27% (78 out of 293) samples of NPS (Supporting Information Figure 3A). RV was detected simultaneously in the three sample sites in 24 of the 137 RV‐positive patients (18%) (Supporting Information Figure 3B). For the purpose of this study of rhinoviruses, all samples that were also positive for EVs by RT‐PCR were excluded from further analyzes: 58 of 74 RV‐positive (78,4%) palatine tonsils, 64 of 97 (66%) RV‐positive adenoids, and 9 of 78 (11.5%) RV‐positive NPS. After applying this exclusion criterion, a total of 55 samples remained RV‐positive and EV‐negative and were included in the downstream analyzes. Sequencing of the 5′UTR RT‐PCR products enabled RV genotyping in 46 samples (Supporting Information Table 1). The analysis of RV genotypes revealed RV of species A in 21/46 (42%), species B in 18/46 (36%), and species C in 11/46 (22%) samples (Supporting Information Table 2). Among the RV genotypes identified, no significant difference was observed between the detection frequencies of RV‐A, RV‐B, and RV‐C (*p*‐value 0.157), likely reflecting the limited number of genotyped samples. Several genotypes of RV species A and B were detected simultaneously in all three sampling sites, and in 11 patients the same RV genotype was detected simultaneously from both tonsil types and corresponding NPS (Supporting Information Table 3). The 5′UTR sequencing strategy used does not allow for accurate genotyping of RV species C, which is a limitation of the study.

### 3.2 In Situ RV Detection by IHC and CISH

**Figure 1:**
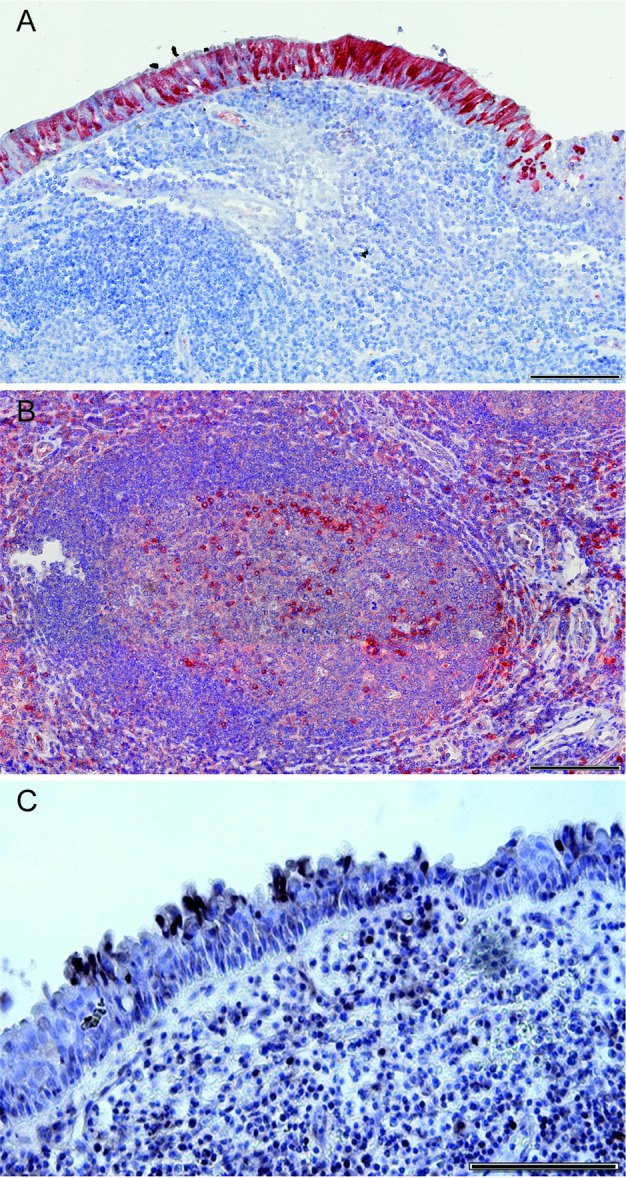
Immunohistochemical staining for RV capsid proteinsVP1 and VP2, and chromogenic in situ hybridization for RV anti‐genome in adenoids. (A) VP1/VP2 staining in pseudostratified ciliated epithelium, with RV‐infected cells indicated by red positive signal; (B) VP1/VP2 staining in cells within a lymphoid follicle and in extrafollicular area; (C) Positive hybridization signal in the epithelium (arrow) and in lymphoid cells within the tissue (arrow heads). Scale bars: 200 µm (A) and (B), and 50 µm (C).

Tonsillar tissues that were positive for RV and negative for EV by RT‐PCR were investigated by IHC for RV capsid proteins VP1 or VP2, and by CISH with a probe specific for the RV anti‐genome (Figure 1). Of 16 palatine tonsils and 33 adenoids tested, respectively 9 (56%) and 20 (60%) were positive by IHC for RV antigen. In adenoids, IHC signal was abundant in the pseudostratified ciliated epithelium (Figure 1A), and also detected, yet less abundantly, in lymphomononuclear cells within lymphoid follicles and in extra‐follicular regions (Figure 1B). In palatine tonsils, RV antigens were detected predominantly in the cytoplasm of cells in lymphoid compartments, both follicular and extra‐follicular, but also in patches of crypt reticulated epithelium and surface squamous epithelium. To confirm the presence of RV replication in tonsils, CISH was done with a probe specific for the anti‐genome on RV‐positive/EV‐negative palatine tonsils and adenoids. Tissues from 13 patients were available for testing with the probe for the RV antigenome: palatine tonsil and adenoid from 3, only palatine tonsil from 4, and only adenoid from 6 patients. All tissues from the 13 RV‐positive/EV‐negative patients were positive by CISH, with signal on the surface epithelia and in lymphoid compartments, both in and out of lymphoid follicles (Figure 1C). RV‐infected and uninfected HeLa cells were included as positive and negative controls in all tested batches, and control tonsillar tissues (n = 6) that tested negative for RV and EV by RT‐PCR were also negative by IHC and CISH (Supporting Information Figure 4).

### 3.3 RV Isolation

To assess the presence of infectious RV in tonsillar tissues and secretions, available samples of tissue macerates and NPS from 17 RV‐positive/EV‐negative patients were inoculated in cell cultures, and RV was recovered from 10 of them (58.8%). The same RV genotypes, RV‐4 and RV‐14, were isolated simultaneously from NPS and adenoid tissues in two patients, while in the remaining eight patients RV was isolated exclusively from adenoid tissues (Table 2).

**Table 2:** Types of RV isolated from adenoids and swabs.

	RV from adenoids			RV from swabs		
Patient	Type	e‐value	Identity	Type	e‐value	Identity
160	RV‐14	7e‐17	98%			
429	RV‐29	4e‐08	100%			
455	RV‐4	2e‐13	96%	RV‐4	2e‐13	96%
490	RV‐14	5e‐69	99%	RV‐14	4e‐70	98%
403	RV‐82	3e‐17	97%			
413	RV‐38	6e‐36	100%			
441	RV‐37	5e‐31	98%			
462	RV‐42	3e‐17	97%			
467	RV‐14	6e‐18	98%			
481	RV‐46	3e‐22	98%			

### 3.4 Cell Types Infected by RV and Tonsillar Cytokine Profile

**Figure 2:**
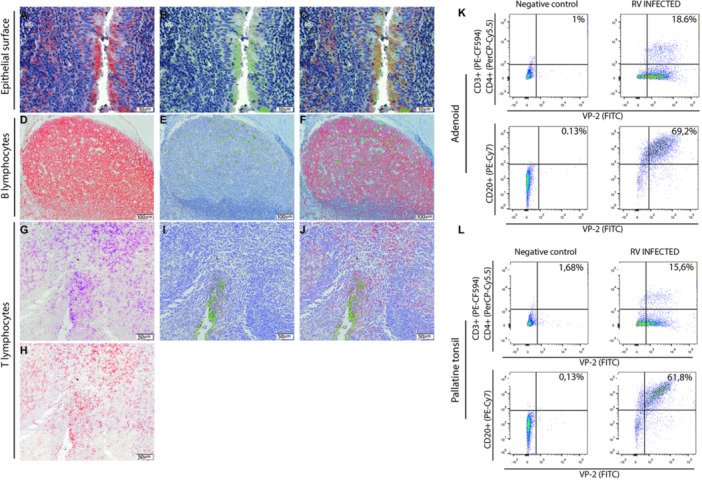
Types of cells infected by RV in adenoids by serial immunohistochemistry for RV proteins VP1/VP2 and markers of cell types. (A) Representative field of an adenoid stained for human cytokeratin, with positive epithelial cells pseudo‐colored in red; (B) Same section as in (A) after erasing the previous staining and restaining for RV VP1/VP2 proteins, with positive cells pseudo‐colored in green; (C) Merge of (A) and (B) confirming that RV VP1/VP2 proteins were detected in epithelial cells; (D) Representative field of an adenoid stained for CD 20, with positive cells pseudo‐colored in red; (E) Same section as in (D) after erasing the previous staining and restaining for RV proteins VP1/VP2, with positive cells pseudo‐colored in green; (F) Merge of (D) and (E), confirming that RV VP1/VP2 proteins were detected in B lymphocytes; (G): Representative field of an adenoid stained for CD 3 with positive cells pseudo‐colored in purple; (H) Same section as in (G), after erasing previous staining and restaining for CD4, with positive cells pseudo‐colored in red; (I) Same section as in (G) and (H) after erasing previous staining and restaining for RV VP1/VP2, with positive cells pseudo‐colored in green; (J) Merge of (H) and (I) confirming that RV VP1/VP2 proteins were detected in CD4 T lymphocytes; (K) and (L) Representative dot plots obtained by flow cytometry respectively from RV‐infected adenoid and palatine tonsil with acquisition of 10^4^ events per assay.

The RV structural protein VP2 was detected in epithelial cells, as evidenced by the serial staining for epithelial cell surface antigen EPCAM (Figure 2A–C). In the parenchyma, sequential IHC showed that follicular VP‐2 positive cells were also positive for CD20 (Figure 2D–F), confirming that RV infects B lymphocytes in human tonsils. Moreover, VP2‐positive cells (Figure 2I) were also positive for CD3 (Figure 2G) and CD4 (Figure 2H), thus confirming that RV infects CD4^+^ T lymphocytes (Figura 2J) in human tonsils in vivo. Of note, RV VP2 was not detected in CD8⁺ or CD11c⁺ cells in our experimental setting (Supporting Information Figure 5), suggesting that RV infection may be specific to certain lympho‐hematopoietic cell types in this kind of tissue, and in our experimental conditions. To further assess cell‐type susceptibility, we performed in vitro infections of TMNCs dissociated from tissues with RV‐16 and RV‐1A, followed by RIFI staining. Again, no RV antigen was detected in CD8⁺ or CD11c⁺ cells, in further support of the findings obtained in naturally infected tissues (Supporting Information Figure 6). Further studies will be needed to verify whether this restriction is generalizable to other immunological settings. Flow cytometry experiments were conducted to determine the relative proportions of different types of RV‐infected cells in the tissues and revealed that approximately 25.57% (median: 23.0%) of CD4 + T lymphocytes and approximately 39.78% (median: 41.55%) of B lymphocytes were positive for RV VP2 (Figure 2J,L), further supporting the data obtained in situ.

**Figure 3:**
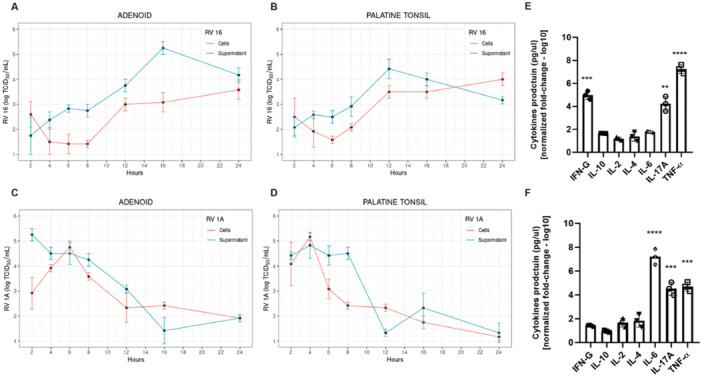
RV progeny production and cytokine profiles in tonsillar mononuclear cells (TMNCs) dissociated from adenoids and tonsils and infected in vitro. TMNCs obtained from adenoids (A and C) and palatine tonsils (B and D) that tested negative for RV natural infection were infected ex vivo with RV‐16 (A and B) or RV‐1A (C and D). Virus titration was done by TCID_50_ in cell pellets and supernatants at the indicated times post infection. Selected cytokines were quantified by a cytometric bead array in supernatants from adenoid TMNCs infected (MOI = 1) with RV‐16 (E) and RV‐1A (F). Results in (E) and (F) are represented as means ± SD of normalized fold‐change based on 10^4^ events per assay. Statistical analyzes were done with GraphPad Prism 6.00 (GraphPad Software, San Diego, California, USA) and significance was considered when p value was less than 0.05.

To obtain further evidence of susceptibility and permissiveness of tonsillar cells to RV, primary cultures of TMNCs dissociated from tonsils negative by real‐time PCR for both RV and EV were infected in vitro with RV‐16 or RV‐1A (MOI = 1) and analyzed 24 h post‐infection by TCID_50_ assay. There was substantial increase in RV‐16 progeny production in primary TMNCs (Figure 3A,B), while titers of RV‐1A progressively decreased by approximately 100‐fold in the first 24 h (Figure 3C,D). Interestingly, both RV genotypes significantly impacted the production of cytokines by TMNCs, albeit in distinct ways. Both RV‐16 and RV‐1A induced significant increase in production of IL‐17 and TNF‐α in TMNCs at 24 h post‐infection (hpi) compared to mock‐inoculated cultures. However, RV‐16 induced a significant secretion of IFN‐γ in addition to IL‐17 and TNF‐α (Figure 3E), whereas RV‐1A significantly induced the secretion of IL‐6 (Figure 3F).

## 4 Discussion

In the present study, RV genome was detected by RT‐PCR in nearly one‐third of adenoids and about one‐quarter of palatine tonsils and NPS from children undergoing tonsillectomy for the treatment of tonsillar hypertrophy or recurrent tonsillitis, in the absence of ARI symptoms for at least 1 month before surgery. The replication of RV in respiratory epithelial cells has been known since our early studies by in situ hybridization of human tissues infected ex vivo and in vivo [11, 12]. Those studies included infection of adenoid explants ex vivo [11] and nasal epithelium from experimentally infected volunteers [12].

This study expands knowledge of RV‐susceptible host cells to include lymphoid cells in both follicular and extrafollicular compartments of naturally infected tonsils, alongside epithelial cells. Unlike a previous finnish study detecting picornaviruses [14], this study confirmed RV‐specific localization by excluding enterovirus‐positive tonsils via RT‐PCR. In addition, the present study showed that RV infects B and CD4⁺ T lymphocytes in tonsils. Remarkably, RV was detected in nearly 40% of the CD20⁺ B lymphocytes from naturally infected tonsils, which may be due to the sheer predominance of B lymphocytes among tonsillar lymphoid cells [15]. In situ hybridization for RV antigenomes strongly supports the presence of RV active replication, not only in epithelial cells, but also in tonsillar lymphomononuclear cells. Further investigation will be needed to uncover the subsets of B and CD4⁺ T lymphocytes that are infected by RV, as well as their stages of maturation.

It is also noteworthy that in 18% of the RV‐positive patients, the virus was detected simultaneously in both tonsil tissues and nasopharyngeal swabs, and sometimes the same genotype of RV was detected in the three sample types, suggesting that tonsils may be sources of shedding of RV into nasopharyngeal secretions. This is also supported by the isolation of RV, including cases with the same RV genotype from tonsillar tissues and nasopharyngeal secretions, confirming that there was production of infectious RV progeny in the tissues. Notably, the infiltration of tonsillar reticulated epithelium by lymphocytes, which is crucial for the initiation of immune responses [16], may conceivably create opportunities also for the passage of RV from lymphoid to polarized epithelial cells, in which the virus may replicate efficiently and produce progeny that appear in secretions. This finding underscores the importance of considering multiple sampling methods to comprehensively assess the prolonged detection of RV and its genotype diversity.

This study did not include follow‐up investigations to assess secondary transmission. Nonetheless, the detection of RV in tonsillar tissues from asymptomatic children raises the possibility that such individuals could serve as sources of silent viral shedding. While the influence of weather and climate on RV epidemiology remains uncertain, the present findings suggest that further studies are needed on the roles of asymptomatic carriers/shedders in the onset of outbreaks in situations that entail exposure of susceptible individuals, such as the beginning of school term in the fall in temperate regions [17, 18]. It should be kept in mind that the present cross‐sectional study design makes it impossible to demonstrate virus persistence, a practical limitation that stems from the impossibility of sampling patients tissues over time.

Genome sequencing revealed the presence of all three rhinovirus species—RV‐A, RV‐B, and RV‐C—in lymphoid tissue. Notably, since CDHR3—the receptor for RV‐C—is poorly expressed in immune cells, the detection of RV‐C RNA may reflect nonspecific uptake, infection of rare susceptible cells, or alternative entry mechanisms. In vitro infection of TMNCs from RV‐negative tonsils showed differential permissiveness, with progeny production by RV‐16 but not by RV‐1A. Accordingly, the cytokine profiles induced by in TMNCs were also different between RV‐16 and RV‐1A, what may be correlated with the difference in viral progeny production, but could also result from dissimilar cell activations by the binding of the viruses to different receptors [18, 19]. Previous studies have shown that high IFN‐α and low RORC2 expressions were associated with RV‐C, while RV‐B was associated with low Tbet and high IFN‐γ expressions [4, 13]. Also, altered expression of inflammatory cytokines by macrophage exposure to different RVs indicated that RV‐16 induced higher expression of CCL20, CCL2, CXCL10, and IL‐10 by macrophages when compared to RV‐1A [18]. It is important to note that the in vitro experiments were done only with RV‐16 and RV‐1A, cautioning against generalization to all rhinoviruses.

Previous in vitro studies done with peripheral blood mononuclear cells provided glimpses at how immune cells respond to RV, with intense proliferation of B cells [20], and activation of CD4 and CD8^+^ T lymphocytes, even in the absence of antigen‐presenting cells [21]. Thus, it is conceivable that RV infection of different types of immune cells may activate them in tonsils. Moreover, RV infection in CD4^+^ T and B lymphocytes in tonsils may interfere with the mounting of local immune responses to different antigens and allergens. In that regard, it is remarkable that adenoid hypertrophy has been associated with an unfavorable course of childhood asthma, and that adenotonsillectomy has been associated with significant reductions in asthma attacks in children [22].

Similar to other persistent viral infections, the prolonged asymptomatic carriage of RV in tonsils could also entail its reactivation in immunosuppressed patients, which befits results from previous studies that showed persistent shedding of RV of the same genotype in children and immunocompromised adults over periods as long as 100 days [23, 24, 25, 26]. The study's cross‐sectional design, with single‐time tissue sampling, limited confirmation of persistent RV infection. Long‐term persistence would require repeated sampling from the same subjects, which is not feasible.

Based on current evidence, the use of RV detection in secretions by RT‐PCR as confirmation of etiology of current respiratory symptoms should be regarded with caution. Early studies based only on virus isolation in cell culture reported infectious RV shedding from asymptomatic subjects ranging from 0.7% to 1.6% in adults and 4.7% in children [27, 28]. Considering only results of RV detection by RT‐PCR in secretions, which is the most readily available routine sample in clinical settings, the rate of positivity in children with tonsillar hypertrophy was close to 12%, which is similar to the RV detection rates in asymptomatic children reported in previous studies in the same city [29]. It is reasonable to consider that RV may stay in tonsillar tissues for prolonged times after an acute infection, which is in agreement with previous data on RV RNA detection by RT‐PCR [30, 31]. Yet, RT‐PCR detection does not necessarily indicate virus replication at the time of sampling.

The clinical relevance of human rhinovirus (RV) in respiratory diseases extends well beyond the common cold [32]. RV infection has been linked to exacerbations and increased morbidity in chronic respiratory diseases such as COPD and asthma, with certain genotypes—particularly RV‐A and RV‐C—associated with more severe outcomes [33]. Co‐infections, including with SARS‐CoV‐2, may further worsen asthma [34], highlighting species‐specific differences in RV pathogenesis [34, 35]. These findings align with our study, which not only identified all three RV species (A, B, and C) in tonsillar tissues from children without acute symptoms, but also demonstrated active infection and replication in CD4⁺ T and B lymphocytes, accompanied by the release of pro‐inflammatory cytokines such as IL‐17, TNF‐α, and IFN‐γ. Although the cross‐sectional design of our study does not permit to establish viral persistence, the presence of RV in immune cells from asymptomatic individuals suggests that tonsillar lymphoid tissues may transiently harbor the virus, what may contribute to the dynamics of RV infection and transmission. In summary, tonsillar lymphoid tissues may be sites of asymptomatic RV replication, and it is conceivable that the consequent immune activation might interfere in recurrent airway inflammation associated with chronic upper airway diseases and allergy.

## Author Contributions

Conceptualization and methodology were carried out by Ronaldo Martins, Flavia E. de Paula, Talita B. G. Mitchell, Miria F. Criado, and Eurico Arruda. Investigation and data curation were performed by Flavia E. de Paula, Talita B. G. Mitchell, Miria F. Criado, Ricardo S. Cardoso, Bruna L. S. Jesus, Italo A. Castro, Murilo Henrique Anzolini Cassiano, Daniela Méria Ramos Rodrigues, Noilson Oliveira, and Lucas Carenzi. Formal analysis and visualization were conducted by Ronaldo Martins, Flavia E. de Paula, Talita B. G. Mitchell, and Miria F. Criado. Resources and clinical support were provided by Lucas Carenzi, Fabiana C. Valera, Edwin Tamashiro, Wilma T. Anselmo‐Lima, and Eurico Arruda. The original draft was written by Ronaldo Martins, and the manuscript was reviewed and edited by Ronaldo Martins, Fabiana C. Valera, Edwin Tamashiro, Wilma T. Anselmo‐Lima, Edwin Tamashiro, and Eurico Arruda. Supervision, project administration, and funding acquisition were overseen by Eurico Arruda.

## Conflicts of Interest

The authors declare no conflicts of interest.

## Supporting information


**Supporting Figure 1:** Study workflow outlining sample collection, processing, and analysis steps. Children aged 3 to 8 years (*n* = 293) undergoing tonsillectomy were enrolled. Tonsillar tissue and nasopharyngeal swabs were collected and processed for RNA extraction, histology, mononuclear cell isolation (TMNCs), and virus isolation. Subsequent analyses included: Detection & Genotyping of rhinovirus (RV) and enterovirus (EV) using RT‐qPCR and sequencing of the 5′ untranslated region (5′ UTR); Histology & Immunohistochemistry (IHC) for detection of RV antigens (VP1/VP2) and cell phenotyping; In Situ Hybridization (ISH) using LNA probes for EV RNA; Flow Cytometry for immunophenotyping of CD3⁺, CD4⁺, and CD20⁺ lymphocytes and intracellular RV proteins; Virus Isolation in HELA‐I and WI38 cells with cytopathic effect (CPE) monitoring and immunofluorescence assay (IFA); and In Vitro Infection of tonsillar mononuclear cells with RV‐1A or RV‐16, followed by measurement of viral titers (TCID₅₀) and cytokine secretion via cytometric bead array (CBA).

**Supporting Figure 2:** Reactivity of the anti‐VP1 antibody with RV‐16 and RV‐1A by immunofluorescence. (A) Uninfected HeLa cells as negative control; (B and C) HeLa cells infected with RV‐1A (B) and HRV‐16 (C) tested with anti‐VP1 antibody. Nuclei staining with DAPI.

**Supporting Figure 3:** Distribution of samples positive for RV in adenoids, palatine tonsils and nasopharyngeal secretions (NPS).

**Supporting Figure 4:** Controls of immuno‐histochemistry (IHC) and colorimetric in situ hybridization (CISH) for RV detection.

**Supporting Figure 5:** Staining for RV proteins VP1/VP2 and the surface markers CD8 and CD14.

**Supporting Figure 6:** Ex vivo infection of tonsillar immune cells with rhinovirus (RV) and analysis of infected cell subsets by immunofluorescence.

Supporting Table 1.

Supporting Table 2.

Supporting Table 3.

## Data Availability

The datasets generated and analyzed in the study are available from the corresponding author upon reasonable request. All relevant data supporting the findings of this study are included in the article and its supplementary information files. The data that support the findings of this study are available from the corresponding author upon reasonable request.
