# Supplementary figures and images for: Rhinovirus Infects B and CD4 T Lymphocytes in Hypertrophic Tonsils in Children
Source: J Med Virol. 2026 Jan 24;98(2):e70809. doi: 10.1002/jmv.70809 (PMC12831225; doi:10.1002/jmv.70809)

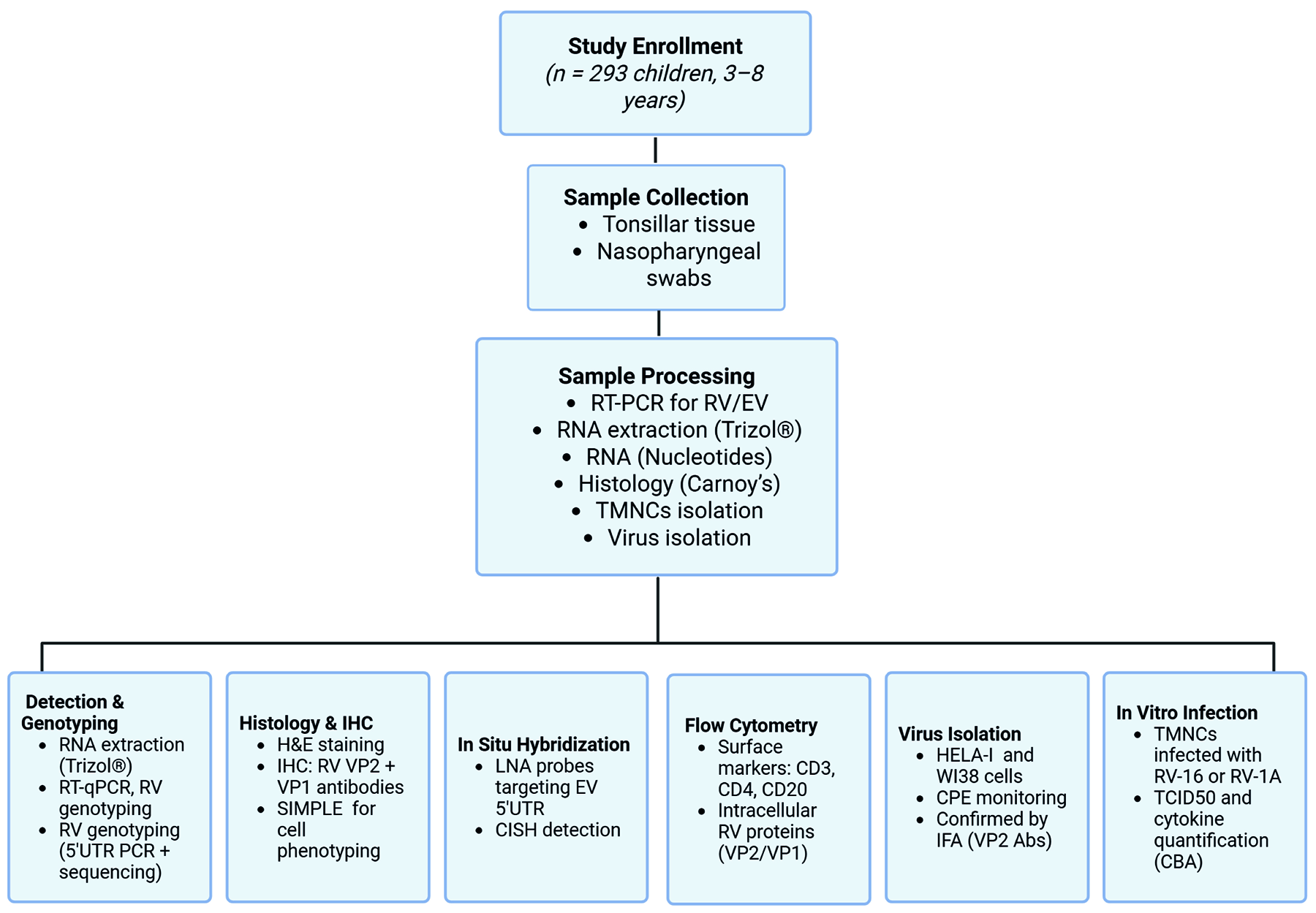

Supplement: Supplementary file 1 — Supporting Figure 1: Study workflow outlining sample collection, processing, and analysis steps. Children aged 3 to 8 years (n = 293) undergoing tonsillectomy were enrolled. Tonsillar tissue and nasopharyngeal swabs were collected and processed for RNA extraction, histology, mononuclear cell isolation (TMNCs), and virus isolation. Subsequent analyses included: Detection & Genotyping of rhinovirus (RV) and enterovirus (EV) using RT‐qPCR and sequencing of the 5′ untranslated region (5′ UTR); Histology & Immunohistochemistry (IHC) for detection of RV antigens (VP1/VP2) and cell phenotyping; In Situ Hybridization (ISH) using LNA probes for EV RNA; Flow Cytometry for immunophenotyping of CD3⁺, CD4⁺, and CD20⁺ lymphocytes and intracellular RV proteins; Virus Isolation in HELA‐I and WI38 cells with cytopathic effect (CPE) monitoring and immunofluorescence assay (IFA); and In Vitro Infection of tonsillar mononuclear cells with RV‐1A or RV‐16, followed by measurement of viral titers (TCID₅₀) and cytokine secretion via cytometric bead array (CBA). [file JMV-98-e70809-s006.tif]

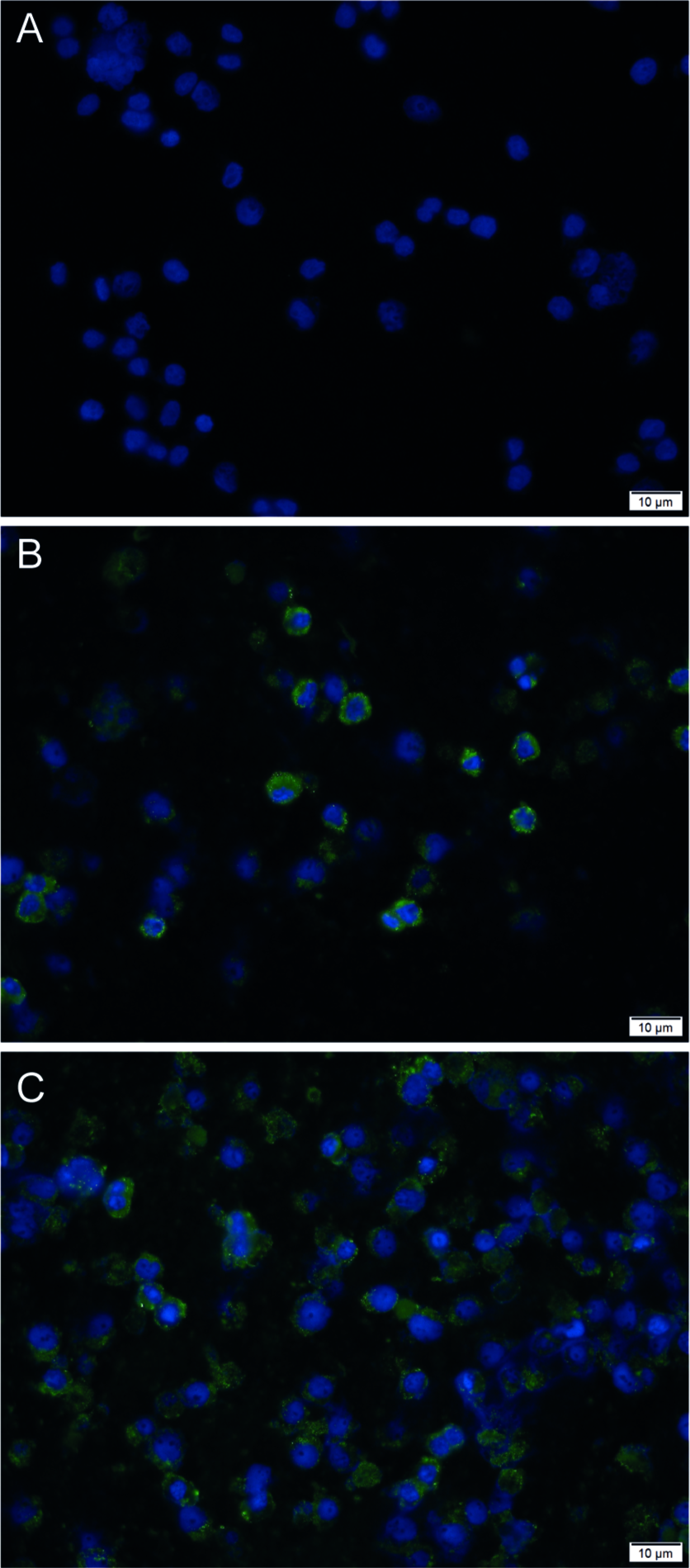

Supplement: Supplementary file 2 — Supporting Figure 2: Reactivity of the anti‐VP1 antibody with RV‐16 and RV‐1A by immunofluorescence. (A) Uninfected HeLa cells as negative control; (B and C) HeLa cells infected with RV‐1A (B) and HRV‐16 (C) tested with anti‐VP1 antibody. Nuclei staining with DAPI. [file JMV-98-e70809-s009.tif]

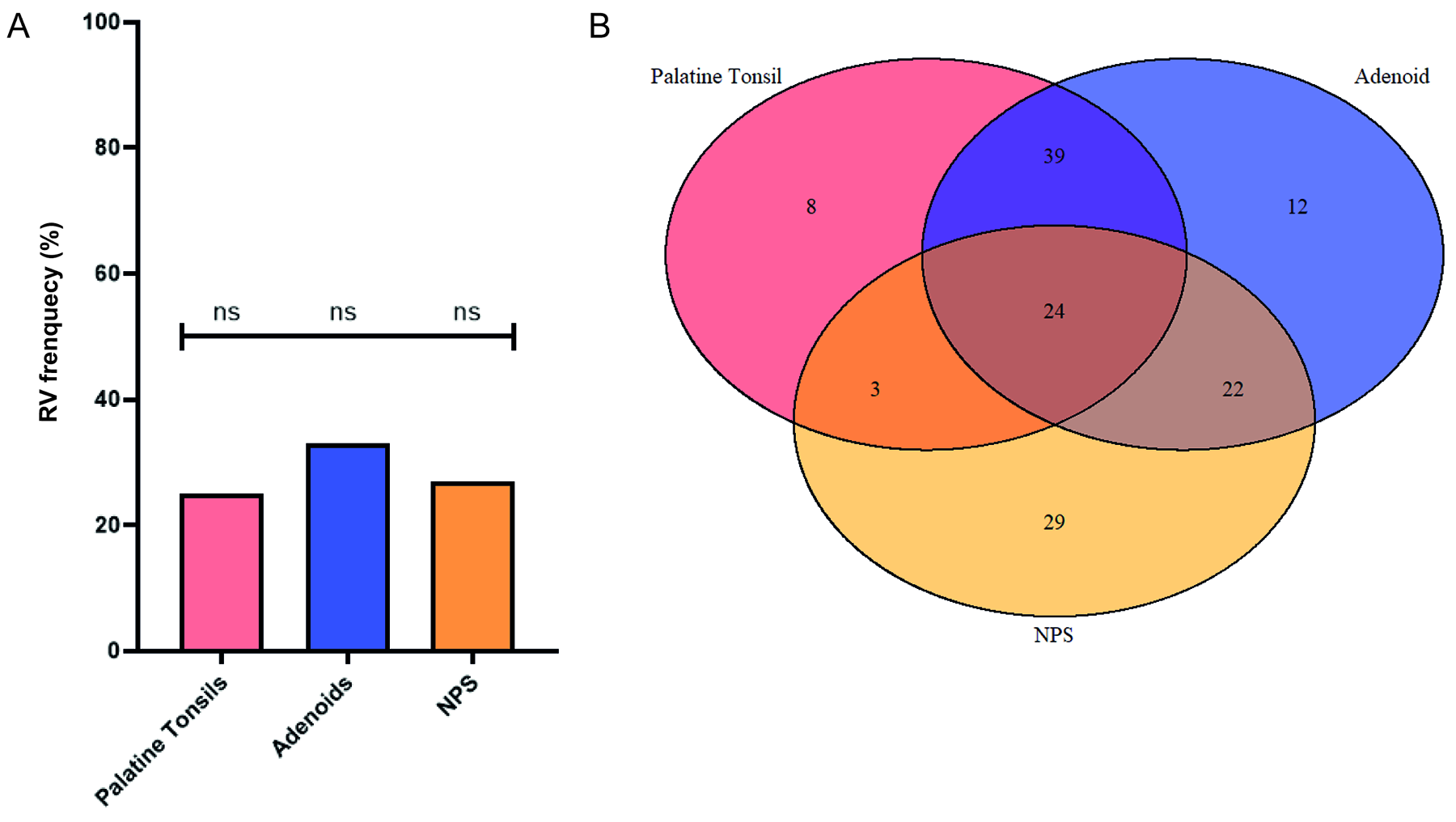

Supplement: Supplementary file 3 — Supporting Figure 3: Distribution of samples positive for RV in adenoids, palatine tonsils and nasopharyngeal secretions (NPS). [file JMV-98-e70809-s004.tif]

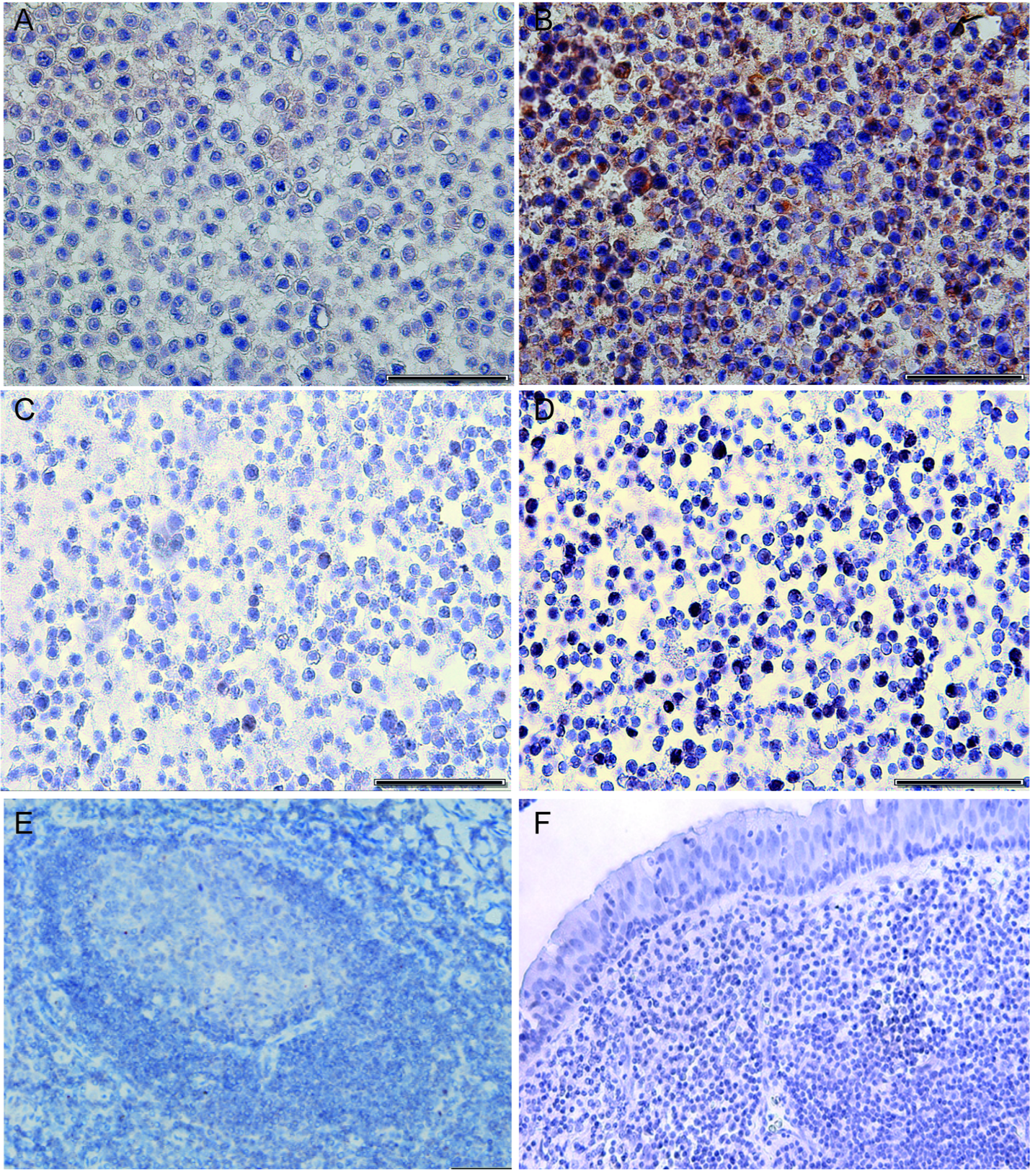

Supplement: Supplementary file 4 — Supporting Figure 4: Controls of immuno‐histochemistry (IHC) and colorimetric in situ hybridization (CISH) for RV detection. [file JMV-98-e70809-s005.tif]

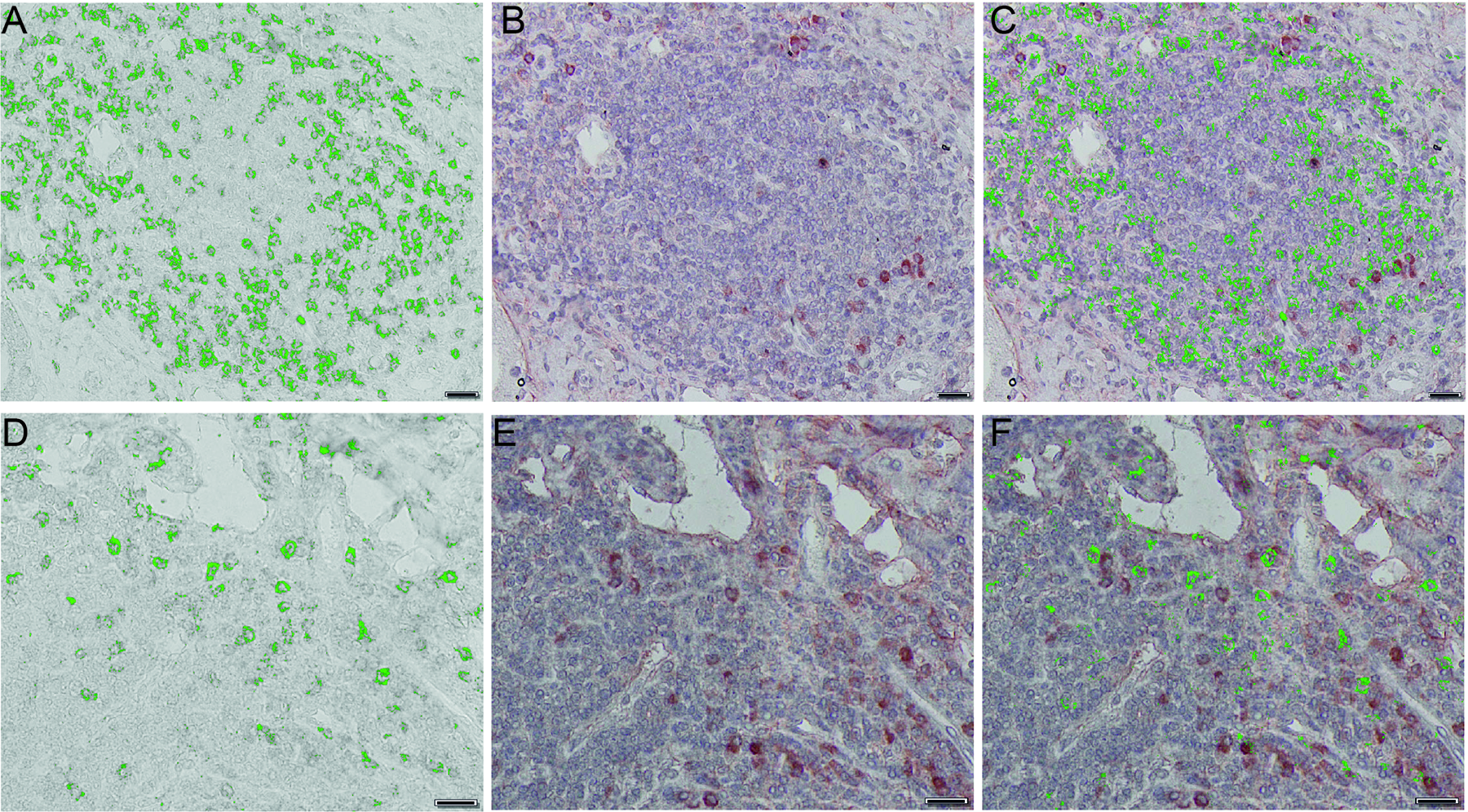

Supplement: Supplementary file 5 — Supporting Figure 5: Staining for RV proteins VP1/VP2 and the surface markers CD8 and CD14. [file JMV-98-e70809-s002.tif]

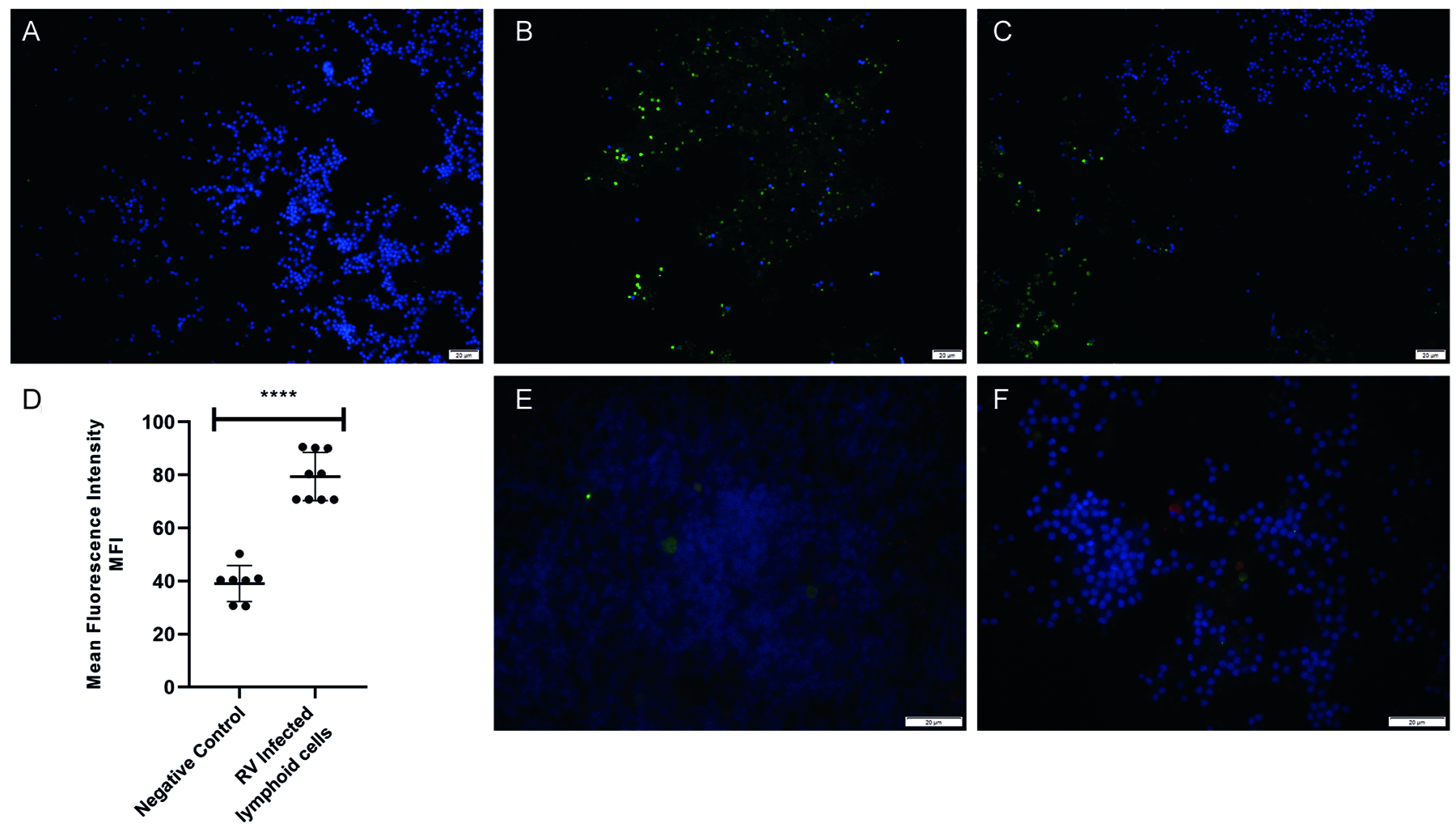

Supplement: Supplementary file 6 — Supporting Figure 6: Ex vivo infection of tonsillar immune cells with rhinovirus (RV) and analysis of infected cell subsets by immunofluorescence. [file JMV-98-e70809-s007.tif]
